# Efficacy of anti-PD-1/PD-L1 plus chemotherapy in treatment-naïve advanced HER2-negative gastric adenocarcinoma among different PD-L1 intervals: an updated meta-analysis and reconstructed individual patient-level data analysis

**DOI:** 10.1186/s12916-026-05124-z

**Published:** 2026-08-08

**Authors:** Yu-Kun Chen, Wen-Wei Wu, Fan Chen, Lan-Jie Chen, Hao-Xiang Wu, Ying-Nan Wang, Ying Jin, Yu-Tong Chen, Zi-Xian Wang

**Affiliations:** 1 Department of Medical Oncology, State Key Laboratory of Oncology in South China, Collaborative Innovation Center for Cancer Medicine, Sun Yat-Sen University Cancer Center, 651 Dongfeng Road East, Guangzhou, 510060 China; 2 Research Unit of Precision Diagnosis and Treatment for Gastrointestinal Cancer, Chinese Academy of Medical Sciences, Guangzhou, 510060 P. R. China; 3 Department of Anesthesiology, State Key Laboratory of Oncology in South China, Guangdong Provincial Clinical Research Center for Cancer, Sun Yat-sen University Cancer Center, Guangzhou, Guangdong 510060 China; 4 Department of Neurosurgery, The First Affiliated Hospital of Sun Yat-sen University, Guangzhou, Guangdong 510080 China; 5 Department of Clinical Research, State Key Laboratory of Oncology in South China, Collaborative Innovation Center for Cancer Medicine, Sun Yat-Sen University Cancer Center, 651 Dongfeng Road East, Guangzhou, 510060 China; 6 Faculty of Medical Science, Jinan University, 601 Huangpu Avenue West, Guangzhou, 510630 China

**Keywords:** Chemo-immunotherapy, Gastric cancer, Combined positive score

## Abstract

**Background:**

Chemo-immunotherapy (CIT) has become the frontline therapy for advanced human epidermal growth factor receptor 2 (HER2)-negative gastric or gastro-esophageal junction adenocarcinoma (GEAC). However, emerging evidence suggests that clinical benefit is primarily derived from patients with a high combined positive score (CPS), whereas the efficacy of CIT among different CPS intervals remained unknown.

**Methods:**

A systemic literature search was performed to screen for RCTs studying addition of PD-1/PD-L1 inhibitors to chemotherapy versus chemotherapy alone in metastatic GEAC with efficacy information across different CPS interval. Pooled analysis of hazard ratios (HRs) for overall survival (OS), progression free survival (PFS), odds ratios (ORs) for objective response rate (ORR) and heterogeneity analysis on treatment efficacy were performed in CPS < 1, 1 ~ 4, 5 ~ 9, ≥10 subgroups.

**Results:**

The analysis showed that adding programmed cell death protein 1/programmed cell death ligand 1 (PD-1/PD-L1) inhibitors to ChT significantly improved overall survival (OS) in the CPS ≥ 10 subgroup (hazard ratio [HR], 0.67 [95%CI 0.61–0.73]), but not in the CPS < 1 or 5–9 subgroups; only a marginal benefit was observed in the CPS 1–4 subgroup. CPS ≥ 10 vs <10 presented the strongest interaction effect between CPS and the efficacy of adding PD-1/PD-L1 inhibitors to ChT (*P*_interaction_ < 0.0001). Additionally, CIT significantly improved the progression-free survival (PFS) and objective response rate (ORR) in both CPS-low (CPS < 1: HR, 0.85 [95%CI 0.72–1.00]; odds ratio [OR], 1.41 [95%CI 1.02–1.96]) and CPS-high (CPS ≥ 10: HR, 0.64 [95%CI 0.58–0.70]; OR, 1.87 [95%CI 1.50–2.33]) subgroups versus ChT alone.

**Conclusions:**

CPS ≥ 10 defines a subgroup deriving the greatest benefit from CIT, supporting its use as a clinically informative threshold for PD-1 inhibitor therapy in advanced GEAC. Nevertheless, patients with CPS < 10 may still gain benefit from CIT when tumor shrinkage is the primary treatment objective.

## Background

Gastric or gastroesophageal junction adenocarcinoma (GEAC) is the fifth most common malignant cancer, accounting for the fourth leading cause of cancer-related mortality [1–3]. Fluoropyrimidine combined with platinum-based chemotherapy (ChT) has long been the standard first-line treatment for metastatic or unresectable GEAC, until the advent of immunotherapy [4–6]. Several large-scale phase III RCTs, including checkmate 649 [4] and KEYNOTE 859 [7], have demonstrated that the addition of PD-1/PD-L1 inhibitors to ChT can significantly improve clinical outcomes. However, when it comes to subgroup analysis by PD-L1 CPS, nivolumab plus ChT only benefits patients with high PD-L1 expression (CPS ≥ 5: HR, 0.70 [95%CI 0.60–0.81]) but not those with low PD-L1 expression (CPS < 5: HR, 0.94 [95%CI 0.78–1.13]). Further, more and more studies revealed that clinical benefit of CIT in intention to treat (ITT) population was mainly derived from high CPS subgroup. A meta-analysis in 2021 concluded that patients with CPS < 5 in CheckMate-649 and CPS < 10 in KEYNOTE-062 could not benefit from chemo-immunotherapy (CIT), even suffered from increased hazard ratio (HR) [8].

To optimize the treatment of GEAC and reduce immune related adverse effects, it is urgent to find out the clinical benefit of immunotherapy among different CPS intervals and recommend treatment strategy personally. In the past year, two meta-analysis suggested CPS = 5 as the best cut-off in usage of immunotherapy [9, 10]. However, both studies focused solely on a single CPS threshold, such as CPS ≥ 1 vs <1, rather than exploring a more elaborate CPS interval, and did not account for interaction effect, which undermined the reliability of their conclusion. It remained uncertain whether patients among CPS between 1 and 9 could benefit from CIT strategy. In recent days, the release of new survival data across different CPS intervals has made more refined analyses possible [11].

In our study, we performed an updated meta-analysis using newly released survival data across different CPS intervals from published phase III RCTs on first-line treatment of advanced gastric and gastroesophageal carcinoma. We applied the inverse variance weighting (IVW) method and further evaluated the clinically informative CPS cut-off score to guide the clinical use of PD-1/PD-L1 inhibitors.

## Methods

A comprehensive search of the PubMed database and the abstracts from annual conferences of the American Society of Clinical Oncology, the European Society of Medical Oncology was conducted from its inception through March 2025. The literature search strategy was provided in supplement data (Additional file 1: Table S1). Additionally, the reference lists of the identified articles were manually checked for any relevant trials. The eligibility and inclusion criteria were as follows: (1) randomized clinical trials (RCTs) that reported HER2-negative GEAC; (2) Randomized phase III trial; (3) Studies investigating the efficacy of anti-PD-1/PD-L1 antibody + standard ChT versus standard ChT alone in the first-line setting; (4) Studies that providing subgroup analysis results using CPS values of 1,5,10 as cut-off points. Exclusion criteria were studies including HER2-positive or previously treated patients, as well as those conducted in the neoadjuvant or adjuvant settings. When encountering duplicate data or subgroup analyses from the same study, the most recent and comprehensive publications were selected for inclusion. Finally, eight publications were selected into our study, including CheckMate-649 [4], KEYNOTE-859 [7], ORIENT-16 [12], RATIONALE-305 [13, 14], KEYNOTE-062 [6], KEYNOTE-590 [5, 15], ATTRACTION-4 [16], GEMSTONE-303 [17]. Data extraction was performed independently by two investigators (Y.K. Chen, W. Zhou) and verified by a third investigator (L.J. Chen). Discrepancies were resolved by consensus.

Unstratified (unless otherwise specified) HRs and odd ratios with 95% CIs reported in the literature were retrieved and pooled to estimate overall treatment effects stratified by CPS (using CPS values of 1,5,10 as cut-off points). Heterogeneity among studies was assessed using Cochran’s Q test and the statistic. In the presence of significant heterogeneity (*P* < 0.10 or *I*^*2*^ > 50%), random-effects meta-analysis models were employed to calculate the pooled HRs. Conversely, fixed-effects models were used when heterogeneity was not significant. To estimate the pooled odds ratio (OR) or hazard ratio (HR) across multiple studies, we applied the inverse variance weighting (IVW) method, a standard fixed-effect meta-analysis approach. This method assigns greater weight to studies with lower variance, ensuring that more precise estimates contribute more significantly to the final pooled estimate. Interaction HR meta-regression analysis was conducted to evaluate the interaction between CPS and anti-PD-1/PD-L1 antibody treatment. All P values were two-sided. Statistical analyses were mainly performed using R 4.4.2 (The R Foundation) with metaphor [18], meta [19], survival and survminer R packages.

### Methods of risk of bias assessment

The risk of bias in the selected trials was independently evaluated by two researchers (Y.K. Chen, L.J. Chen), utilizing the Cochrane Risk of Bias 2 (RoB2) tool for randomized controlled trials. The initial assessment revealed that the methodological quality of the included trials was categorized as having a low risk of bias. (Additional file 2: Figure S1).

### Reconstruction of Time-to-event outcome

To further validate the prognostic stratification ability of CPS in both the chemo-immunotherapy and chemotherapy groups, we adopted a graphical reconstructive algorithm to estimate time-to-event outcomes from reported KM plots [20, 21]. Hazard ratios were reconstructed from marginal Cox proportional hazards model. Reconstructed KM plots were compared to original plots by checking the visual shape, marginal HR, log-rank values, median OS, PFS and number-at-risk tables.

### Sensitivity analyses

To evaluate the robustness and stability of the pooled estimates, several sensitivity analyses were performed. First, leave-one-out analyses were conducted by sequentially excluding each individual trial and recalculating the pooled effect estimates for OS, PFS, and ORR to assess the influence of any single study on the overall results (Additional file 3: Tables S2–S4). Second, pooled estimates obtained using random-effects models were compared with those derived from fixed-effect models to evaluate the impact of between-study heterogeneity on the main findings (Additional file 4: Table S5). Consistency in the direction and magnitude of treatment effects across these analyses was considered supportive of the robustness of the pooled results.

## Results

### Study Characteristics

**Fig. 1:**
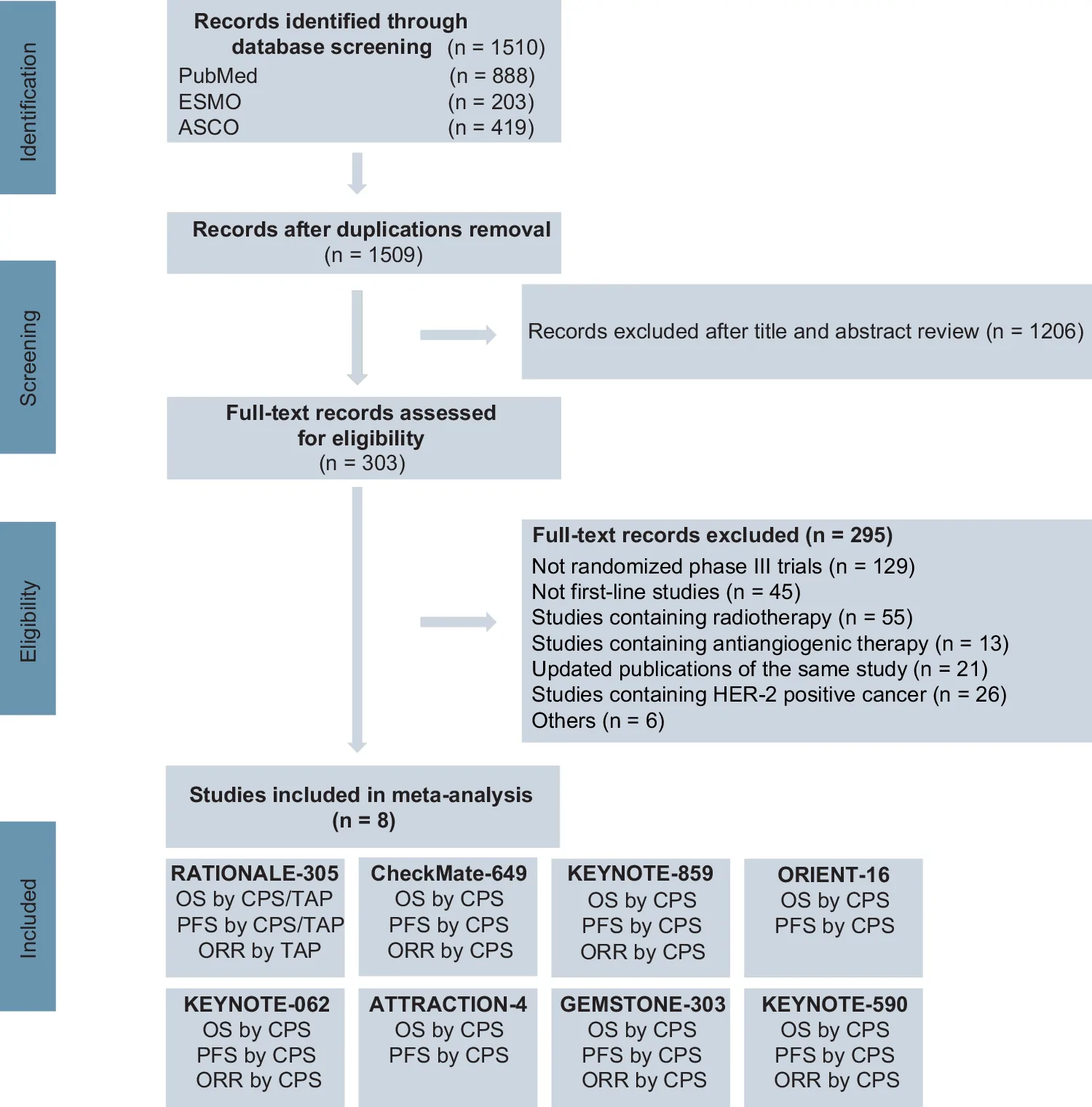
PRISMA flowchart of study inclusions and exclusions. CPS, combined positive score; HER2, human epidermal growth factor receptor 2; PD-1, programmed cell death 1; PD-L1, programmed cell death ligand 1

**Fig. 2:**
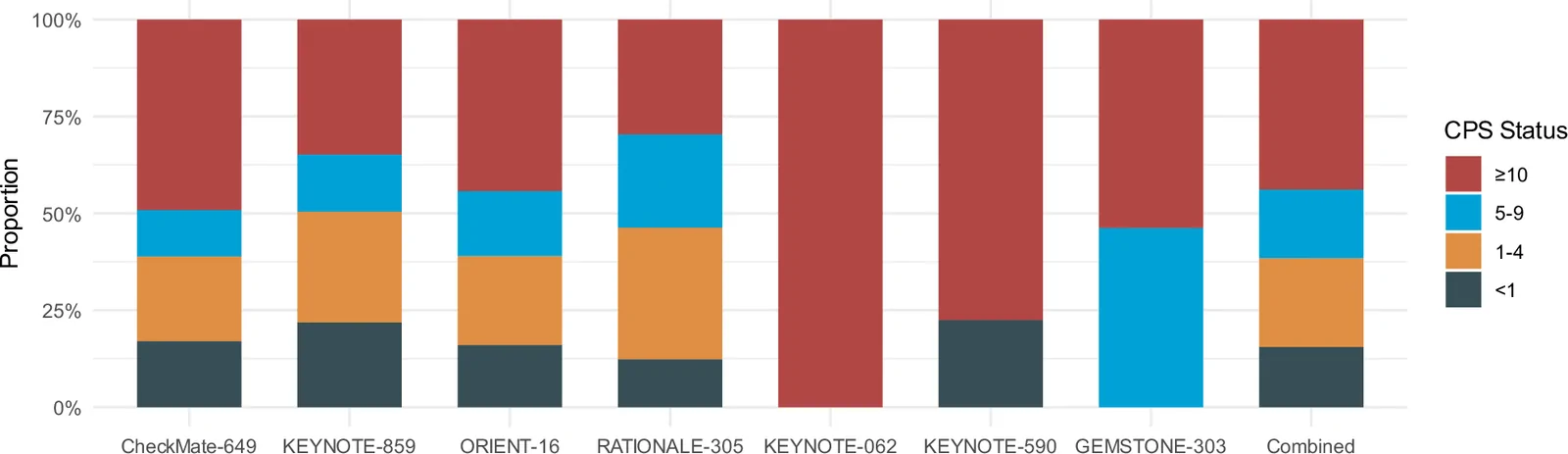
Proportion of different CPS intervals among eight randomized clinical trials

The initial search identified a total of 1510 potentially relevant studies, among which eight randomized controlled trials met the predefined inclusion criteria after comprehensive screening and evaluation, including CheckMate-649, KEYNOTE-859, ORIENT-16, RATIONALE-305, KEYNOTE-062, KEYNOTE-590, ATTRACTION-4, GEMSTONE-303 (Fig. 1; Table 1). Collectively, these trials enrolled 6718 patients with advanced gastric or gastroesophageal junction cancer, with 3366 patients allocated to the CIT group and 3352 patients to the ChT group. Among the ChT regimens administered, 5728 patients received oxaliplatin-based ChT and 990 patients received cisplatin-based ChT. In terms of PD-L1 expression, 861(15.5%) patients were classified as CPS < 1, 1273(22.9%) patients as CPS 1 ~ 4, 983(17.7%) patients as CPS 5 ~ 9, and 2440(43.9%) patients as CPS ≥ 10 (Fig. 2). All trials contributed to OS and PFS analysis.CheckMate-649, KEYNOTE-859, ORIENT-16, KEYNOTE-062, ATTRACTION-4, and GEMSTONE-303 provided data for ORR analysis, while CheckMate-649 and ORIENT-16 with K-M curves in different CPS subgroups were used for individual participant data reconstruction analysis [20].

**Table 1:** Baseline characteristics of potentially eligible trials

Trial, Year of Publication	Arm	Patients, No.	Patients With CPS status, No. (%)	Treatment	PD-L1 assay	Included or Not
CheckMate 649: Lancet (2021)	Test arm	789	781 (99)	Nivolumab plus XELOX, once every three weeks **OR** Nivolumab plus FOLFOX, once every two weeks	28-8 pharmDx assay (Dako Agilent)	Yes
	Control arm	792	780 (98)	Placebo plus XELOX, once every three weeks **OR** Placebo plus FOLFOX, once every two weeks		
ORIENT 16: JAMA (2023)	Test arm	327	327 (100)	Sintilimab plus XELOX, once every three weeks	22C3 pharmDx assay (Dako Agilent)	Yes
	Control arm	323	323 (100)	Placebo plus XELOX, once every three weeks		
KEYNOTE 859: Lancet Oncol (2023)	Test arm	790	790 (100)	Pembrolizumab plus TF, once every three weeks **OR** Pembrolizumab plus XELOX, once every three weeks	22C3 pharmDx assay (Dako Agilent)	Yes
	Control arm	789	789 (100)	Placebo plus TF, once every three weeks **OR** Placebo plus XELOX, once every three weeks		
RATIONALE 305: ESMO (2023)	Test arm	501	491 (98)	Tislelizumab plus TF, once every three weeks **OR** Tislelizumab plus XELOX, once every three weeks	SP263 assay (Ventana Roche)	Yes
	Control arm	496	483 (97)	Placebo plus TF, once every three weeks **OR** Placebo plus XELOX, once every three weeks		
KEYNOTE 062: JAMA Oncol(2020)	Test arm 1	256	256 (100)	Pembrolizumab, once every three weeks	22C3 pharmDx assay (Dako Agilent)	Yes
	Test arm 2	257	257 (100)	Pembrolizumab plus TF, once every three weeks		
	Control arm	250	250 (100)	Placebo plus TF, once every three weeks		
ATTRACTION 4:Lancet Oncol(2022)	Test arm	362	362(100)	Nivolumab plus SOX, once every three weeks **OR** Nivolumab plus CAPOX, once every three weeks	28-8 pharmDx assay (Dako Agilent)	Yes
	Control arm	362	362(100)	Placebo plus SOX, once every three weeks **OR** Placebo plus CAPOX, once every three weeks		
KEYNOTE 590:Lancet(2021)^a^	Test arm	99	97 (98)	Pembrolizumab plus TF, once every three weeks	22C3 pharmDx assay (Dako Agilent)	Yes
	Control arm	102	100(98)	Placebo plus TF, once every three weeks		
GEMSTONE-303: JAMA (2025)	Test arm	241	241 (100)	Sugemalimab plus CAPOX, once every three weeks	SP263 assay (Ventana Roche)	Yes
	Control arm	238	238 (100)	Placebo plus CAPOX, once every three weeks		

*HER2* human epidermal growth factor receptor 2, *XELOX/CAPOX* oxaliplatin/capecitabine, *FOLFOX* fluorouracil/leucovorin/oxaliplatin, *TF* cisplatin/fluorouracil, *SOX* S-1/oxaliplatin

^a^Only esophageal adenocarcinoma subgroup in KEYNOTE-590 was included

### Effect of CPS interval on OS benefit with chemo-immunotherapy

**Fig. 3:**
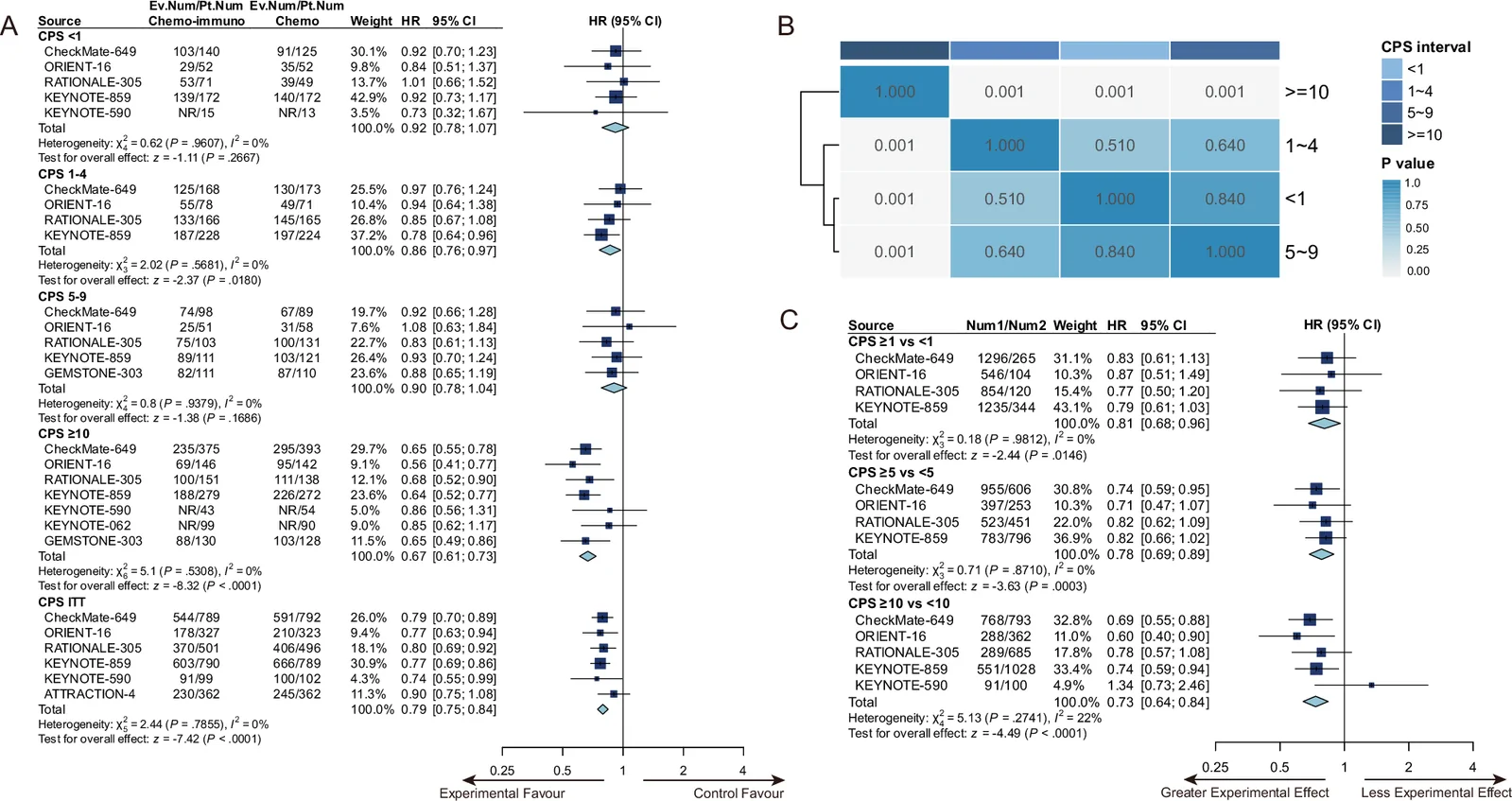
**A** Forest plots showing the HR for overall survival of PD-1/PD-L1 inhibitors plus chemotherapy versus chemotherapy alone among different CPS intervals; (**B**) Efficacy heterogeneity analysis between population among different CPS intervals; P value is represented by color and labels in the rectangle. **C** Interactive term analysis of overall survival between treatment and CPS scores in condition of different CPS cut-offs. *NR, not reported*

CIT combination therapy led to significant survival benefit compared to ChT alone in all random assigned population (pooled HR, 0.79 [95%CI 0.75–0.84]; *P* < 0.0001). No significant inter-trial heterogeneity was observed (*P* = 0.7855, *I*^*2*^ = 0%) (Fig. 3A). To evaluate the clinical benefit of the CIT strategy across different CPS subgroups, a pooled analysis was performed among CPS ≥ 10, CPS 5–9, CPS 1–4, and CPS < 1 intervals. The results demonstrated a significant improvement on OS in the CPS ≥ 10 subgroup (HR, 0.67 [95%CI 0.61–0.73]; *P* < 0.0001), modest effects in CPS 1–4 (HR, 0.86 [95%CI 0.76–0.97]; *P* = 0.0180), and no significant benefit in CPS < 1 or 5–9 (HR, 0.92 [95%CI 0.78–1.07]; *P* = 0.2667, and 0.90 [95%CI 0.78–1.04]; *P* = 0.1686, respectively). Further, efficacy heterogeneity analysis revealed that the CPS ≥ 10 subgroup differed significantly from the CPS < 1, 1–4, and 5–9 subgroups (all P values = 0.0010), while no significant heterogeneity was observed among the latter three subgroups (Fig. 3B). The interaction effect between CPS and the efficacy of adding PD-1/PD-L1 inhibitors to ChT gradually increased across CPS thresholds of 1, 5, and 10, with the strongest interaction observed at CPS = 10 (*P*_interaction_ value = 0.0146, =0.0003, <0.0001, respectively; Fig. 3C).

### Effect of CPS interval on PFS benefit with chemo-immunotherapy

**Fig. 4:**
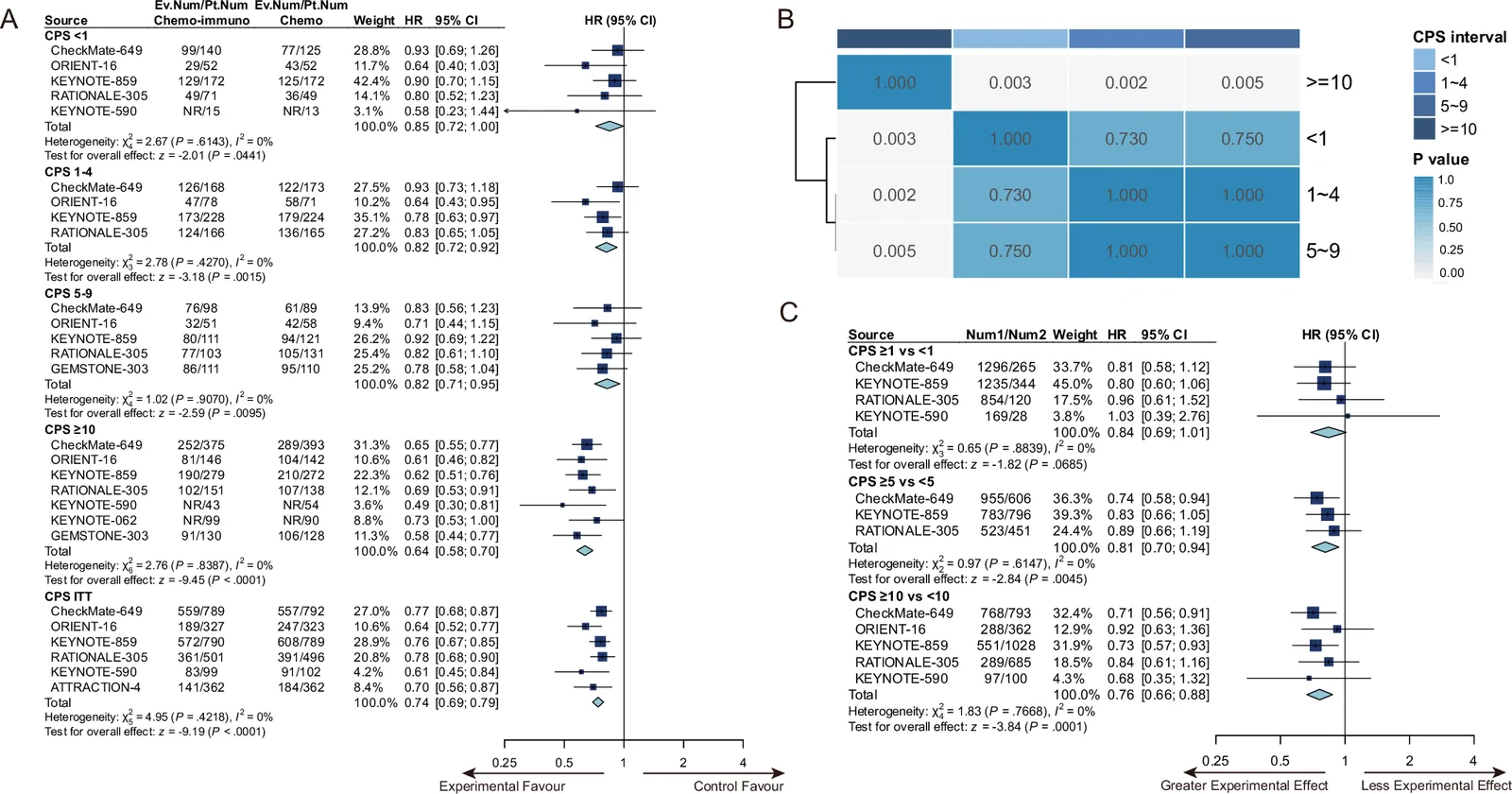
**A** Forest plots showing the HR for progression free survival of PD-1/PD-L1 inhibitors plus chemotherapy versus chemotherapy alone among different CPS intervals; (**B**) Efficacy heterogeneity analysis between population among different CPS intervals; P value is represented by color and labels in the rectangle. **C** Interactive term analysis of progression free survival between treatment and CPS scores in condition of different CPS cut-offs. *NR, not reported*

CIT brought a pooled 26% reduction in the risk of progression compared to ChT alone in randomly assigned population (HR, 0.74 [95%CI 0.69–0.79]; *P* < 0.0001), and no significant inter-trial heterogeneity was observed (*P* = 0.4218, I^2^ = 0%) (Fig. 4A). A significant improvement on PFS was observed across the CPS ≥ 10, 5–9, 1–4, <1 subgroups, with HRs of 0.64 (95% CI, 0.58–0.70; *P* < 0.0001), 0.82 (95% CI, 0.71–0.95; *P* = 0.0095), 0.82 (95% CI, 0.72–0.92; *P* = 0.0015), and 0.85 (95%CI, 0.72–1.00; *P* = 0.0441), respectively. Notably, the magnitude of PFS benefit gradually attenuated with decreasing CPS expression, with the most pronounced benefit observed in the CPS ≥ 10 subgroup. Further, efficacy heterogeneity analysis revealed the CPS ≥ 10 subgroup differed significantly from the CPS < 1, 1–4, and 5–9 subgroups (all *P* values < 0.01) (Fig. 4B). The interaction effect between CPS and the efficacy of adding PD-1/PD-L1 inhibitors to ChT gradually increased across CPS thresholds of 1, 5, and 10, with the strongest interaction observed at CPS = 10. (*P*_interaction_ value = 0.0685, =0.0045, =0.0001, respectively; Fig. 4C).

### Effect of CPS interval on ORR with chemo-immunotherapy

**Fig. 5:**
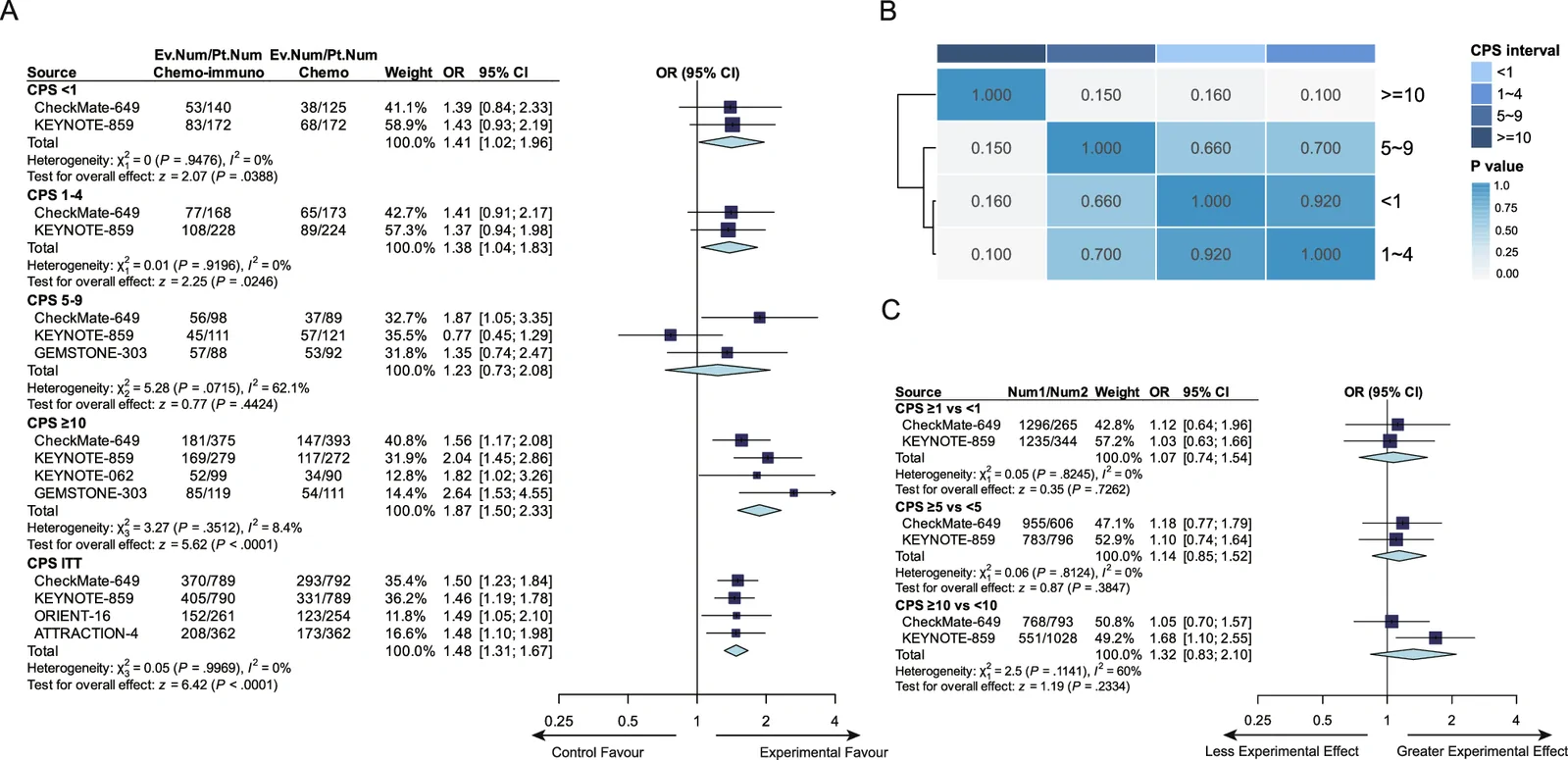
**A** Forest plots showing the OR for objective response rate of PD-1/PD-L1 inhibitors plus chemotherapy versus chemotherapy alone among different CPS intervals; (**B**) Efficacy heterogeneity analysis between population among different CPS intervals; P value is represented by color and labels in the rectangle. **C** Interactive term analysis of objective response rate between treatment and CPS scores in condition of different CPS cut-offs

In ORR analysis, CIT combination therapy still led to significant survival benefit compared to ChT alone in all randomly assigned population (pooled OR, 1.48 [95%CI 1.31–1.67]; *P* < 0.0001), and no significant inter-trial heterogeneity was observed (*P* = 0.9969, I^2^ = 0%). CIT significantly improved response rates not only in the CPS ≥ 10 subgroup (OR, 1.87 [95%CI 1.50–2.33]; P < 0.0001) but also in CPS < 1 (OR, 1.41 [95%CI 1.02–1.96]; *P* = 0.0388) and CPS 1–4 (OR, 1.38 [95%CI 1.04–1.83]; *P* = 0.0246) (Fig. 5A). Nevertheless, CIT only showed marginal benefit in CPS low subpopulation compared to CPS ≥ 10 subgroup. Notably, efficacy heterogeneity analysis across CPS subgroups showed no significant difference (Fig. 5B). Interaction analysis showed no association between CPS thresholds and ORR benefit (All *P*_interaction_ value > 0.1), indicating consistent response improvements with CIT across CPS intervals (Fig. 5C).

#### Clinical benefit of immunotherapy rather than chemotherapy was determined by CPS scores

**Fig. 6:**
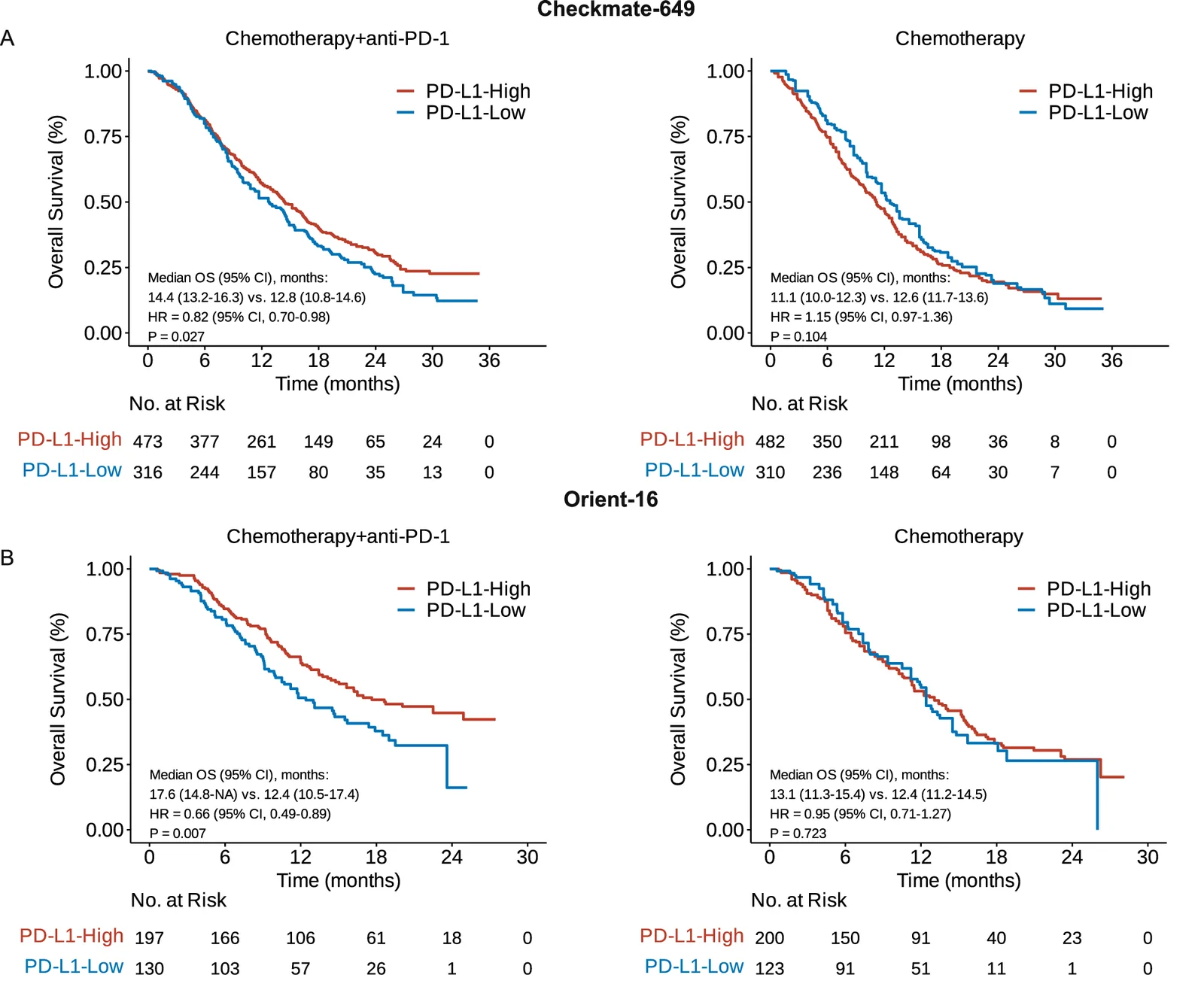
Reconstructed individual patient survival data of (**A**) CheckMate-649 (**B**) Orient-16 and stratified by PD-L1 expression

To further investigate whether CPS primarily impacts the efficacy of CIT rather than ChT alone, we stratified the intention-to-treat populations from CheckMate-649 and ORIENT-16 trials by CPS ≥ 10. In CheckMate-649, the patients with higher PD-L1 expression presented much better survival benefit than patients with PD-L1 low expression in CIT subgroup (HR, 0.82 [95%CI 0.70–0.98]; *P* = 0.0270), no benefit was observed in ChT subgroup (HR, 1.15 [95%CI 0.97–1.36]; *P* = 0.1040) (Fig. 6A). Consistent with CheckMate-649, the patients with high PD-L1 expression receiving chemo-immunotherapy in Orient-16 also presented much better survival benefit than patients with low PD-L1 expression (HR, 0.66 [95%CI 0.49–0.89]; *P* = 0.0070), but not in those receiving ChT alone (HR, 0.95 [95%CI 0.71–1.27]; *P* = 0.7230) (Fig. 6B).

## Discussion

Previous studies, including CheckMate-649 and KEYNOTE-859, demonstrated the efficacy of adding PD-1/PD-L1 inhibitors to ChT in ITT population. However, in PD-L1 low subgroups (CPS < 1), the benefit was limited. In CheckMate-649, significant interactions were observed for CPS < 5 versus ≥ 5 in OS (P = 0.009) and PFS (P = 0.02), but not for CPS < 1 versus ≥ 1. While CPS ≥ 1 showed a survival benefit, this was primarily attributable to patients with high PD-L1 expression [22]. Reflecting these findings, the 2025 NCCN guidelines now recommends pembrolizumab, nivolumab, tislelizumab as first-line options for CPS ≥ 5 gastric and GEJ cancers (Category I) [23].

Our updated meta-analysis clearly confirmed that addition of immunotherapy to ChT provides the most pronounced overall survival benefit in patients with a CPS ≥ 10, whereas no clear benefit was observed in patients with CPS < 1. However, a more detailed meta-analysis based on stratified CPS intervals revealed that patients with a CPS of 1–4 derived only marginal benefit, while those with a CPS of 5–9 did not experience a statistically significant survival advantage from combination therapy. In other words, among patients with a CPS of 1 ~ 10, the clinical benefit of CIT was non-linear, and efficacy did not increase proportionally with higher CPS scores. One possible explanation was that other factors had interfered with the predictive performance of CPS, such as microsatellite instability (MSI) status, another factor that closely correlated with tumor mutation burden [24]. MSI-high patients distributed among different CPS subgroups, accounting for some patients responding to CIT combination therapy with low PD-L1 expression [25, 26]. What’s more, some emerging indicators, including EBV status, TMB, are also reported to be associated with efficacy of CIT combination [27–30]. Owing to limitations in the available source data, we could not evaluate whether potential confounders, including TMB and MSI status, influenced the therapeutic efficacy of chemoimmunotherapy in patients with low CPS expression. More effective predictors are needed to optimize the usage of CIT strategy in low PD-L1 expression population. Another reason was that bias derived from different PD-L1 detection methods led to the variation of efficacy estimation, especially in subpopulation with marginal PD-L1 expression. Lacking consensus on PD-L1 detection limited comparability of PD-L1 interpretation between different centers, inspiring us to establish a consensus framework for PD-L1 detection and scoring criteria [31]. To address the heterogeneity across different CPS intervals, efficacy heterogeneity analysis of OS and PFS further confirmed the similarity among the CPS < 1, 1–4 and 5–9 subgroups, suggesting that the survival benefit observed in the ITT population receiving CIT might primarily be attributed to the CPS ≥ 10 subpopulation.

What’s more, our study demonstrates a statistically significant improvement in PFS and ORR among PD-L1 low subpopulation after addition of PD-1/PD-L1 inhibitors to ChT. These findings may suggest that some PD-L1 low patients could still derive short-term clinical benefit from chemo-immunotherapy, particularly in scenarios where rapid tumor shrinkage or disease control is considered clinically important. Although the CPS < 10 versus CPS ≥ 10 stratification demonstrated the most pronounced trend of interaction with chemo-immunotherapy, ORR data were available from only a limited number of studies, and no statistically significant interaction between CPS status and ORR benefit was observed. Therefore, we consider these findings to be hypothesis-generating. Importantly, ORR and OS outcomes may reflect distinct biological processes. ORR and PFS mainly captures early tumor cytoreduction, which may be substantially influenced by the chemotherapy backbone and transient treatment-related immune activation [32, 33]. In contrast, durable OS benefit likely depends more heavily on sustained anti-tumor immune surveillance and long-term immunologic memory, which may be less effectively established in low-CPS tumors with relatively limited pre-existing immune activation [34, 35]. Taken together, our findings support CPS ≥ 10 as a clinically informative cutoff associated with the greatest and most consistent survival benefit from PD-1/PD-L1 inhibitor-based chemo-immunotherapy in advanced GEAC.

Previous meta-analyses had primarily assessed the benefit of chemo-immunotherapy using only single CPS cutoff values, such as CPS ≥ 1 or ≥ 5, and did not take interaction effect into consideration [9, 10]. As a result, the observed statistical significance in these subgroups was often driven by the therapeutic benefit seen in patients with CPS ≥ 10 and the conclusions were unreliable. In contrast, our study is the first to precisely evaluate the efficacy of chemo-immunotherapy across distinct CPS intervals. We found that patients with CPS < 1, 1–4, and 5–9 exhibited comparable therapeutic responses, suggesting a degree of efficacy homogeneity within these groups. Importantly, our analysis identified CPS ≥ 10 as the clinically informative threshold for distinguishing patients who derive a clear survival benefit from combination therapy.

For patients with low PD-L1 expression, more intensive strategies should be taken, and more effective targets needed to be discovered for a better survival. Lin S. et al reported cadonilimab plus ChT as first line therapy for non-HER2 positive advanced gastric or gastroesophageal junction adenocarcinoma in COMPASSION-15 trial. In the patients with a PD-L1 CPS < 5, the median OS was 13.7 months (95%CI 11.5–17.5) in the cadonilimab group and 11.4 months (95%CI 10.1–13.0) in the placebo group (HR, 0.75 [95%CI 0.56–1.00]) [36]. The median PFS was 6.9 months (95% CI 5.7–9.0) with cadonilimab and 4.6 months (95% CI 4.3–5.6) with placebo (HR,0.60 [95%CI 0.45–0.79]). Cadonilimab, PD-1/CTLA-4 bispecific antibody, showed promising efficacy in CPS low GEAC, indicating that CTLA4 signaling blockade might contribute to CPS low population [37]. Nevertheless, COMPASSION-15 trial didn’t disclose the clinical benefit of cadonilimab plus ChT in CPS < 1 population, which was important for the clinical decision in PD-L1 negative population. Another emerging promising target, CLDN18.2, shows a pronouncing effect in CLDN18.2 + GEAC. The results from SPOTLIGHT suggested that target CLDN18.2 with zolbetuximab significantly improved OS and PFS in combination with mFOLFOX6 compared to placebo plus mFOLFOX6 in patients with CLDN18.2-positive, non-HER2 positive, locally advanced unresectable or metastatic GEAC [38]. In KYM901 trial, D.Y. Ruan et al. reported CMG901 had an objective response rate of 28% and a disease control rate of 63% in gastric or gastro-oesophageal junction cancer, regardless of previous lines of therapy and anti-PD-1/PD-L1 exposure [39]. What’s more, some phase II trials also presented several promising strategies in PD-L1 negative or low expression population, including regorafenib with nivolumab and ChT (mPFS 13.0 m [95%CI 7.6-NR]; mOS: Not reached) [40], DKN-01 in combination with tislelizumab and ChT (mPFS: 11.3 m[95%CI 5.75–12.02]; mOS: 19.5 m[95%CI 15.2–24.4]) as first line therapy in untreated advanced gastric or gastroesophageal junction adenocarcinoma [41]. As reported by Samuel et al., the objective response rate showed no significant difference between PD-L1 positive and PD-L1 negative populations (77% [95%CI 46–95] vs 75% [95%CI 48–93]; *P* > 0.99) in the single arm study of regorafenib combined with nivolumab and ChT. What’s more, DKN-01 combined with tislelizumab and ChT also presented promising efficacy in PD-L1 low population (ORR between CPS ≥ 5 and CPS < 5: 67% [95%CI 22.3–95.7] vs 86% [95%CI 57.2–98.2]).

However, there are several limitations to our study. First, we didn’t discuss whether the choice of PD-L1 scoring methods could affect the results, for only a few trials adopted TPS or TAP as indicators with available efficacy information among different TPS/TAP intervals. Nevertheless, V. Formica et al. recently pointed out that TPS might not be a preferred selector for anti-PD-1/PD-L1 treatment in GEAC [9]. According to RATIONALE 305 reported on ESMO 2024, TAP showed concordance with CPS on overall agreement. No significant survival benefit was observed in TAP low subgroups, including TAP < 1% (HR, 0.98 [95%CI 0.64–1.50]), TAP < 5% (HR, 0.91 [95%0.74–1.12], and TAP < 10% (HR, 0.91 [95%CI 0.77–1.07]). In contrast, only patients with TAP ≥ 10% demonstrated a clear survival advantage from the addition of a PD-1 inhibitor to ChT (HR, 0.57 [95%CI 0.43–0.76]), further supporting our conclusion [14]. Second, our study mainly relied on published results to infer clinical benefit in different CPS intervals through inverse variance weighting (IVW). We must acknowledge that the lack of individual patient data would affect the accuracy to some extent. What’s more, several methodological limitations should also be acknowledged. First, the HRs included in this meta-analysis were derived from a mixture of stratified and unstratified Cox models, as harmonized estimates based on a uniform model specification were not consistently available from the published reports. Second, heterogeneity across the included trials may also arise from differences in trial design, patient populations, geographic composition, chemotherapy backbones, immune checkpoint inhibitors, and so on. To evaluate the robustness of the findings, we performed sensitivity and leave-one-out analyses, as summarized in Additional file 3: Tables S2–S5. Therefore, while the pooled estimates provide an overall synthesis of the available evidence, their magnitude should be interpreted with appropriate caution, particularly for analyses with residual heterogeneity.

In conclusion, our findings suggest that a CPS ≥ 10 serves as the most appropriate threshold for guiding the use of PD-1/PD-L1 inhibitors in GEAC, identifying the subgroup most likely to derive meaningful clinical benefit. Nonetheless, given the observed improvement in objective response rates with the addition of PD-1/PD-L1 inhibitors to ChT compared to ChT alone, this combination may still be considered for patients with low PD-L1 expression, particularly in cases where rapid tumor shrinkage or symptom relief is the primary therapeutic objective.

## Conclusions

CPS ≥ 10 defines a subgroup deriving the greatest benefit from CIT, supporting its use as a clinically informative threshold for PD-1 inhibitor therapy in advanced GEAC. Nevertheless, patients with CPS < 10 may still gain benefit from CIT when tumor shrinkage is the primary treatment objective.

## Supplementary information

Additional file 1: Table S1. Search Information.

Additional file 2: Figure S1. Risk of bias assessment.

Additional file 3: Tables S2–S4.

Additional file 4. Table S5. Comparison of random and fixed effect model.

## Data Availability

The dataset supporting the conclusions of this article is included within the article and its additional files. Raw data and code required to produce the major results of this paper could be requested with reasonable reason by contact corresponding author.

## Abbreviations

CIT: chemo-immunotherapy
HER-2: human epidermal growth factor receptor 2
GEAC: gastric or gastro-esophageal junction adenocarcinoma
CPS: combined positive score
HR: hazard ratio
OS: overall survival
PFS: progression free survival
OR: odds ratio
ORR: objective response rate
ChT: chemotherapy
ITT: intention-to-treat
IVW: inverse variance weighting
RCT: randomized clinical trial
RoB2: Cochrane Risk of Bias 2
MSI: microsatellite instability

## References

[1] Thrift AP, Wenker TN, El-Serag HB. Global burden of gastric cancer: epidemiological trends, risk factors, screening and prevention. Nat Rev Clin Oncol. 2023;20:338–49. 10.1038/s41571-023-00747-0 36959359

[2] Mamun TI, Younus S, Rahman MH. Gastric cancer-Epidemiology, modifiable and non-modifiable risk factors, challenges and opportunities: An updated review. Cancer Treat Res Commun. 2024;41:100845. 10.1016/j.ctarc.2024.100845 39357127

[3] Sung H, Ferlay J, Siegel RL, Laversanne M, Soerjomataram I, Jemal A, et al. Global cancer statistics 2020: GLOBOCAN estimates of incidence and mortality worldwide for 36 cancers in 185 countries. CA Cancer J Clin. 2021;71:209–49. 10.3322/caac.21660 33538338

[4] Janjigian YY, Shitara K, Moehler M, Garrido M, Salman P, Shen L, et al. First-line nivolumab plus chemotherapy versus chemotherapy alone for advanced gastric, gastro-oesophageal junction, and oesophageal adenocarcinoma (CheckMate 649): a randomised, open-label, phase 3 trial. Lancet. 2021;398:27–40. 10.1016/S0140-6736(21)00797-2 PMC8436782 34102137

[5] Sun JM, Shen L, Shah MA, Enzinger P, Adenis A, Doi T, et al. Pembrolizumab plus chemotherapy versus chemotherapy alone for first-line treatment of advanced oesophageal cancer (KEYNOTE-590): a randomised, placebo-controlled, phase 3 study. Lancet. 2021;398:759–71. 10.1016/S0140-6736(21)01234-4 34454674

[6] Shitara K, Van Cutsem E, Bang YJ, Fuchs C, Wyrwicz L, Lee KW, et al. Efficacy and safety of pembrolizumab or pembrolizumab plus chemotherapy vs chemotherapy alone for patients with first-line, advanced gastric cancer: The KEYNOTE-062 Phase 3 randomized clinical trial. JAMA Oncol. 2020;6:1571–80. 10.1001/jamaoncol.2020.3370 PMC7489405 32880601

[7] Rha SY, Oh DY, Yanez P, Bai Y, Ryu MH, Lee J, et al. Pembrolizumab plus chemotherapy versus placebo plus chemotherapy for HER2-negative advanced gastric cancer (KEYNOTE-859): a multicentre, randomised, double-blind, phase 3 trial. Lancet Oncol. 2023;24:1181–95. 10.1016/S1470-2045(23)00515-6 37875143

[8] Zhao JJ, Yap DWT, Chan YH, Tan BKJ, Teo CB, Syn NL, et al. Low programmed Death-Ligand 1-Expressing subgroup outcomes of first-line immune checkpoint inhibitors in gastric or esophageal adenocarcinoma. J Clin Oncol. 2022;40:392–402. 10.1200/JCO.21.01862 34860570

[9] Formica V, Morelli C, Fornaro L, Riondino S, Rofei M, Fontana E, et al. PD-L1 thresholds predict efficacy of immune checkpoint inhibition in first-line treatment of advanced gastroesophageal adenocarcinoma. A systematic review and meta-analysis of seven phase III randomized trials. ESMO Open. 2024;9:103967. 10.1016/j.esmoop.2024.103967 PMC11613432 39541621

[10] Li JB, Lai MY, Lin ZC, Guan WL, Sun YT, Yang J, et al. The optimal threshold of PD-L1 combined positive score to predict the benefit of PD-1 antibody plus chemotherapy for patients with HER2-negative gastric adenocarcinoma: a meta-analysis. Cancer Immunol Immunother. 2024;73:132. 10.1007/s00262-024-03726-1 PMC11098986 38753055

[11] Lemery S Meeting of the Oncologic Drugs Advisory Committee Meeting Announcement https://www.fda.gov/advisory-committees/advisory-committee-calendar/september-26-2024-meeting-oncologic-drugs-advisory-committee-meeting-announcement-09262024: U.S. Food & Drug Administration; 2024 [

[12] Xu J, Jiang H, Pan Y, Gu K, Cang S, Han L, et al. Sintilimab plus chemotherapy for unresectable gastric or gastroesophageal junction cancer: The ORIENT-16 randomized clinical trial. JAMA. 2023;330:2064–74. 10.1001/jama.2023.19918 PMC10698618 38051328

[13] Qiu MZ, Oh DY, Kato K, Arkenau T, Tabernero J, Correa MC, et al. Tislelizumab plus chemotherapy versus placebo plus chemotherapy as first line treatment for advanced gastric or gastro-oesophageal junction adenocarcinoma: RATIONALE-305 randomised, double blind, phase 3 trial. BMJ. 2024;385:e078876. 10.1136/bmj-2023-078876 38806195

[14] Moehler M, Oh D-Y, Kato K, Tabernero J, Cruz-Correa M, Wyrwicz LS, et al. Tislelizumab (TIS) plus chemotherapy (CT) vs placebo (PBO) plus CT in HER2-negative advanced or metastatic gastric or gastro-esophageal junction adenocarcinoma (GC/GEJC): PD-L1 biomarker analysis from RATIONALE-305. Annals of Oncology. 2024;35:S162–S204.

[15] Metges JP, Kato K, Sun JM, Shen L, Enzinger PC, Adenis A, et al. Pembrolizumab plus chemotherapy versus chemotherapy for advanced esophageal cancer: 5-year extended follow-up for the randomized phase III KEYNOTE-590 study. ESMO Open. 2025;10:105854. 10.1016/j.esmoop.2025.105854 PMC12670541 41259897

[16] Kang YK, Chen LT, Ryu MH, Oh DY, Oh SC, Chung HC, et al. Nivolumab plus chemotherapy versus placebo plus chemotherapy in patients with HER2-negative, untreated, unresectable advanced or recurrent gastric or gastro-oesophageal junction cancer (ATTRACTION-4): a randomised, multicentre, double-blind, placebo-controlled, phase 3 trial. Lancet Oncol. 2022;23:234–47. 10.1016/S1470-2045(21)00692-6 35030335

[17] Zhang X, Wang J, Wang G, Zhang Y, Fan Q, Lu C, et al. First-Line Sugemalimab Plus Chemotherapy for Advanced Gastric Cancer: The GEMSTONE-303 Randomized Clinical Trial. JAMA. 2025;333:1305–14. 10.1001/jama.2024.28463 PMC11851304 39992668

[18] Viechtbauer W. Conducting meta-analyses in R with the metafor package. Journal of Statistical Software. 2010;36:1–48.

[19] Schwarzer G, Carpenter JR, Rücker G Meta-Analysis with R: Springer Cham; 2015 16 October.

[20] Guyot P, Ades AE, Ouwens MJ, Welton NJ. Enhanced secondary analysis of survival data: reconstructing the data from published Kaplan-Meier survival curves. BMC Med Res Methodol. 2012;12:9. 10.1186/1471-2288-12-9 PMC3313891 22297116

[21] Zhao JJ, Syn NL, Tan BKJ, Yap DWT, Teo CB, Chan YH, Sundar R. KMSubtraction: reconstruction of unreported subgroupsurvival data utilizing published Kaplan-Meier survival curves. BMC Med Res Methodol. 2022;22(1):93. 10.1186/s12874-022-01567-z PMC8978435 35369867

[22] Shah MA, Kennedy EB, Alarcon-Rozas AE, Alcindor T, Bartley AN, Malowany AB, et al. Immunotherapy and targeted therapy for advanced gastroesophageal cancer: ASCO guideline. J Clin Oncol. 2023;41:1470–91. 10.1200/JCO.22.02331 36603169

[23] Ajani JA, D’Amico TA, Bentrem DJ, Corvera CU, Das P, Enzinger PC, et al. Gastric cancer, version 2.2025, NCCN clinical practice guidelines in oncology. J Natl Compr Canc Netw. 2025;23:169–91. 10.6004/jnccn.2025.0022 40341199

[24] Yoon HH, Jin Z, Kour O, Kankeu Fonkoua LA, Shitara K, Gibson MK, et al. Association of PD-L1 expression and other variables with benefit from immune checkpoint inhibition in advanced gastroesophageal cancer: systematic review and Meta-analysis of 17 Phase 3 randomized clinical trials. JAMA Oncol. 2022;8:1456–65. 10.1001/jamaoncol.2022.3707 PMC9412834 36006624

[25] Chao J, Fuchs CS, Shitara K, Tabernero J, Muro K, Van Cutsem E, et al. Assessment of pembrolizumab therapy for the treatment of microsatellite instability-high gastric or gastroesophageal junction cancer among patients in the KEYNOTE-059, KEYNOTE-061, and KEYNOTE-062 clinical trials. JAMA Oncol. 2021;7:895–902. 10.1001/jamaoncol.2021.0275 PMC8017478 33792646

[26] Sun H, Nered S, Tryakin A, Abgaryan M, Artamonova E, Stroganova A, et al. Microsatellite instability (MSI) in patients with gastric cancer (GC) and correlation with PD-L1 expression. J Clin Oncol. 2024;42:389.

[27] Qiu MZ, Wang C, Wu Z, Zhao Q, Zhao Z, Huang CY, et al. Dynamic single-cell mapping unveils Epstein‒Barr virus-imprinted T-cell exhaustion and on-treatment response. Signal Transduct Target Ther. 2023;8:370. 10.1038/s41392-023-01622-1 PMC10514267 37735150

[28] Jang JY, Jeon Y, Jeong SY, Lim SH, Kang WK, Lee J, et al. The optimal tumor mutational burden cutoff value as a novel marker for predicting the efficacy of programmed cell death-1 checkpoint inhibitors in advanced gastric cancer. J Gastric Cancer. 2023;23:476–86. 10.5230/jgc.2023.23.e29 PMC10412974 37553133

[29] Bai Y, Xie T, Wang Z, Tong S, Zhao X, Zhao F, et al. Efficacy and predictive biomarkers of immunotherapy in Epstein-Barr virus-associated gastric cancer. J Immunother Cancer. 2022;10:e004080. 10.1136/jitc-2021-004080 PMC8896035 35241494

[30] Lima A, Sousa H, Medeiros R, Nobre A, Machado M. PD-L1 expression in EBV associated gastric cancer: a systematic review and meta-analysis. Discov Oncol. 2022;13:19. 10.1007/s12672-022-00479-0 PMC8941030 35318527

[31] Ahn S, Kim KM. PD-L1 expression in gastric cancer: interchangeability of 22C3 and 28-8 pharmDx assays for responses to immunotherapy. Mod Pathol. 2021;34:1719–27. 10.1038/s41379-021-00823-9 34002009

[32] Zitvogel L, Apetoh L, Ghiringhelli F, Kroemer G. Immunological aspects of cancer chemotherapy. Nat Rev Immunol. 2008;8:59–73. 10.1038/nri2216 18097448

[33] Chen DS, Mellman I. Oncology meets immunology: the cancer-immunity cycle. Immunity. 2013;39:1–10. 10.1016/j.immuni.2013.07.012 23890059

[34] Kaufman HL, Schwartz LH, William WN Jr., Sznol M, Fahrbach K, Xu Y, et al. Evaluation of classical clinical endpoints as surrogates for overall survival in patients treated with immune checkpoint blockers: a systematic review and meta-analysis. J Cancer Res Clin Oncol. 2018;144:2245–61. 10.1007/s00432-018-2738-x PMC11813378 30132118

[35] Plesca I, Tunger A, Muller L, Wehner R, Lai X, Grimm MO, et al. Characteristics of tumor-infiltrating lymphocytes prior to and during immune checkpoint inhibitor therapy. Front Immunol. 2020;11:364. 10.3389/fimmu.2020.00364 PMC7064638 32194568

[36] Shen L, Zhang Y, Li Z, Zhang X, Gao X, Liu B, et al. First-line cadonilimab plus chemotherapy in HER2-negative advanced gastric or gastroesophageal junction adenocarcinoma: a randomized, double-blind, phase 3 trial. Nat Med. 2025. 10.1038/s41591-024-03450-4 39843940

[37] Pang X, Huang Z, Zhong T, Zhang P, Wang ZM, Xia M, et al. Cadonilimab, a tetravalent PD-1/CTLA-4 bispecific antibody with trans-binding and enhanced target binding avidity. MAbs. 2023;15:2180794. 10.1080/19420862.2023.2180794 PMC10012886 36872527

[38] Shitara K, Lordick F, Bang YJ, Enzinger P, Ilson D, Shah MA, et al. Zolbetuximab plus mFOLFOX6 in patients with CLDN18.2-positive, HER2-negative, untreated, locally advanced unresectable or metastatic gastric or gastro-oesophageal junction adenocarcinoma (SPOTLIGHT): a multicentre, randomised, double-blind, phase 3 trial. Lancet. 2023;401:1655–68. 10.1016/S0140-6736(23)00620-7 37068504

[39] Ruan DY, Liu FR, Wei XL, Luo SX, Zhuang ZX, Wang ZN, et al. Claudin 18.2-targeting antibody-drug conjugate CMG901 in patients with advanced gastric or gastro-oesophageal junction cancer (KYM901): a multicentre, open-label, single-arm, Phase 1 trial. Lancet Oncol. 2025;26:227–38. 10.1016/S1470-2045(24)00636-3 39788133

[40] Cytryn SL, Moy RH, Cowzer D, Shah RH, Chou JF, Joshi SS, et al. First-line regorafenib with nivolumab and chemotherapy in advanced oesophageal, gastric, or gastro-oesophageal junction cancer in the USA: a single-arm, single-centre, phase 2 trial. Lancet Oncol. 2023;24:1073–82. 10.1016/S1470-2045(23)00358-3 PMC11208000 37666264

[41] Klempner SJ, Sonbol MB, Wainberg ZA, Uronis HE, Chiu VK, Scott AJ, et al. DKN-01 in Combination with tislelizumab and chemotherapy as first-line therapy in advanced gastric or gastroesophageal junction adenocarcinoma: DisTinGuish. J Clin Oncol. 2025;43:339–49. 10.1200/JCO.24.00410 PMC11771358 39432867

