# Supplementary material for: Efficacy of anti-PD-1/PD-L1 plus chemotherapy in treatment-naïve advanced HER2-negative gastric adenocarcinoma among different PD-L1 intervals: an updated meta-analysis and reconstructed individual patient-level data analysis
Source: BMC Med. 2026 Aug 8;24:617. doi: 10.1186/s12916-026-05124-z (PMC13737756; doi:10.1186/s12916-026-05124-z)
Supplement: Supplementary file 1 [file 12916_2026_5124_MOESM2_ESM.pdf]

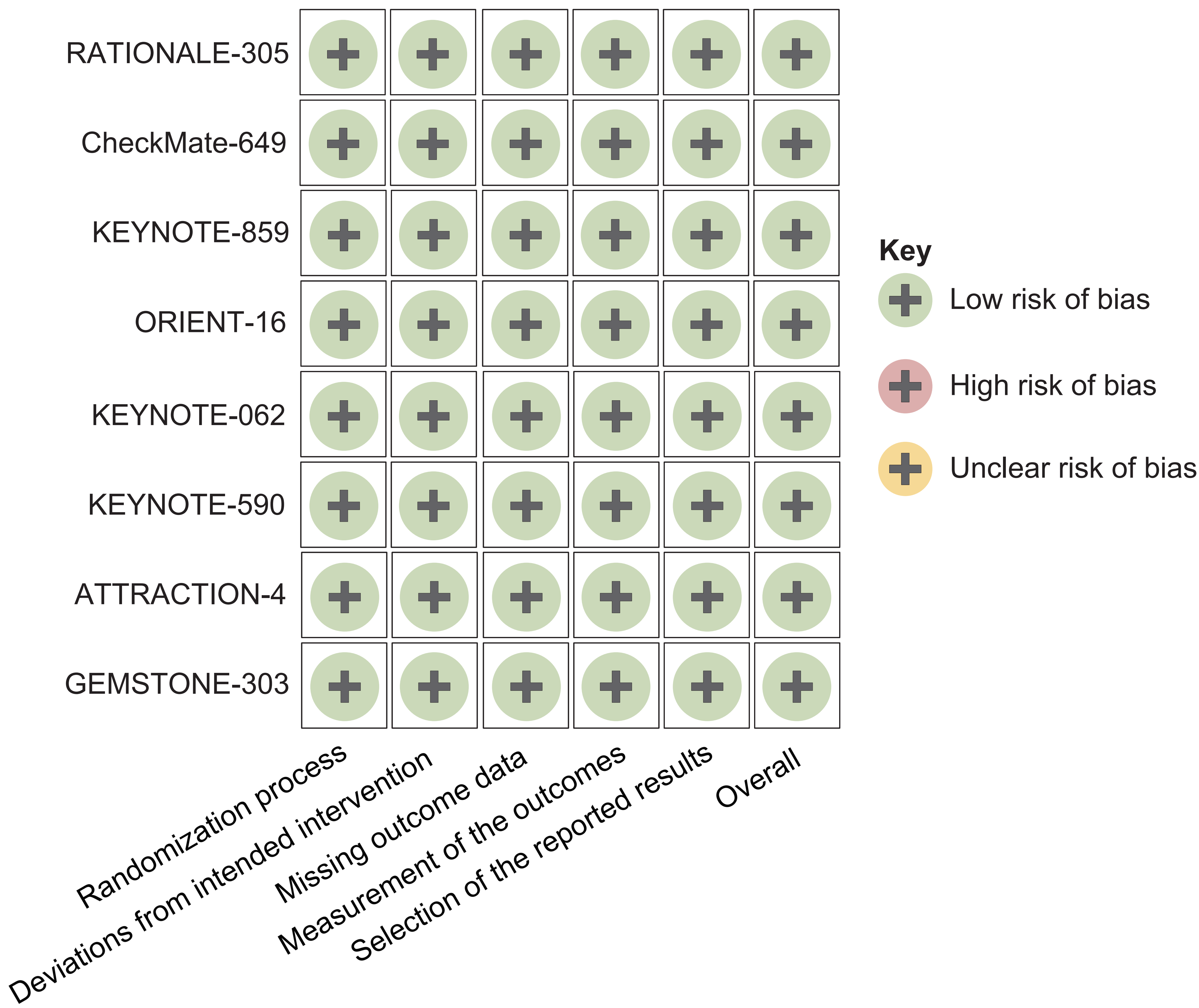

RATIONALE-305
CheckMate-649
KEYNOTE-859
ORIENT-16
KEYNOTE-062
KEYNOTE-590
ATTRACTION-4
GEMSTONE-303
Randomization process
Deviations from intended intervention
Missing outcome data
Measurement of the outcomes
Selection of the reported results
Overall
Key
Low risk of bias
High risk of bias
Unclear risk of bias
