# Supplementary material for: Efficacy of anti-PD-1/PD-L1 plus chemotherapy in treatment-naïve advanced HER2-negative gastric adenocarcinoma among different PD-L1 intervals: an updated meta-analysis and reconstructed individual patient-level data analysis
Source: BMC Med. 2026 Aug 8;24:617. doi: 10.1186/s12916-026-05124-z (PMC13737756; doi:10.1186/s12916-026-05124-z)
Supplement: Supplementary file 2 [file 12916_2026_5124_MOESM1_ESM.docx]

| **Supplementary Table 1: Search Information** | |
| --- | --- |
| Date of search | 1969/01/01-2025/03/01 |
| Database | PubMed, ASCO, ESMO |
| PubMed | ((("gastro esophageal"[All Fields] OR "gastro oesophageal"[All Fields] OR "gastro oesophagitis"[All Fields] OR "gastroesophagal"[All Fields] OR "gastroesophageal"[All Fields] OR "gastroesophagic"[All Fields] OR "gastroesophagitis"[All Fields] OR "gastrooesophageal"[All Fields]) AND ("cancer s"[All Fields] OR "cancerated"[All Fields] OR "canceration"[All Fields] OR "cancerization"[All Fields] OR "cancerized"[All Fields] OR "cancerous"[All Fields] OR "neoplasms"[MeSH Terms] OR "neoplasms"[All Fields] OR "cancer"[All Fields] OR "cancers"[All Fields])) OR (("esophagogastric junction"[MeSH Terms] OR ("esophagogastric"[All Fields] AND "junction"[All Fields]) OR "esophagogastric junction"[All Fields] OR ("gastroesophageal"[All Fields] AND "junction"[All Fields]) OR "gastroesophageal junction"[All Fields]) AND ("cancer s"[All Fields] OR "cancerated"[All Fields] OR "canceration"[All Fields] OR "cancerization"[All Fields] OR "cancerized"[All Fields] OR "cancerous"[All Fields] OR "neoplasms"[MeSH Terms] OR "neoplasms"[All Fields] OR "cancer"[All Fields] OR "cancers"[All Fields])) OR ("stomach neoplasms"[MeSH Terms] OR ("stomach"[All Fields] AND "neoplasms"[All Fields]) OR "stomach neoplasms"[All Fields] OR ("gastric"[All Fields] AND "cancer"[All Fields]) OR "gastric cancer"[All Fields])) AND ("randomized controlled trial"[Publication Type] OR "randomized controlled trials as topic"[MeSH Terms] OR "randomized clinical trial"[All Fields] OR "randomised clinical trial"[All Fields]) AND ("immune checkpoint inhibitors"[Pharmacological Action] OR "immune checkpoint inhibitors"[Supplementary Concept] OR "immune checkpoint inhibitors"[All Fields] OR "pd 1 inhibitor"[All Fields] OR "immune checkpoint inhibitors"[MeSH Terms] OR ("immune"[All Fields] AND "checkpoint"[All Fields] AND "inhibitors"[All Fields]) OR ("immune checkpoint inhibitors"[Pharmacological Action] OR "immune checkpoint inhibitors"[Supplementary Concept] OR "immune checkpoint inhibitors"[All Fields] OR "pd l1 inhibitor"[All Fields] OR "immune checkpoint inhibitors"[MeSH Terms] OR ("immune"[All Fields] AND "checkpoint"[All Fields] AND "inhibitors"[All Fields]) OR ("pd"[All Fields] AND "l1"[All Fields] AND "inhibitor"[All Fields])) OR ("immune checkpoint inhibitors"[Pharmacological Action] OR "immune checkpoint inhibitors"[Supplementary Concept] OR "immune checkpoint inhibitors"[All Fields] OR "immune checkpoint inhibitor"[All Fields] OR "immune checkpoint inhibitors"[MeSH Terms] OR ("immune"[All Fields] AND "checkpoint"[All Fields] AND "inhibitors"[All Fields]) OR ("immune"[All Fields] AND "checkpoint"[All Fields] AND "inhibitor"[All Fields])) OR ("chemotherapy s"[All Fields] OR "drug therapy"[MeSH Terms] OR ("drug"[All Fields] AND "therapy"[All Fields]) OR "drug therapy"[All Fields] OR "chemotherapies"[All Fields] OR "drug therapy"[MeSH Subheading] OR "chemotherapy"[All Fields])) AND ((("first"[All Fields] OR "firsts"[All Fields]) AND "line"[All Fields]) OR ("metastasi"[All Fields] OR "neoplasm metastasis"[MeSH Terms] OR ("neoplasm"[All Fields] AND "metastasis"[All Fields]) OR "neoplasm metastasis"[All Fields] OR "metastasis"[All Fields]) OR ("recurrance"[All Fields] OR "recurrence"[MeSH Terms] OR "recurrence"[All Fields] OR "recurrences"[All Fields] OR "recurrencies"[All Fields] OR "recurrency"[All Fields] OR "recurrent"[All Fields] OR "recurrently"[All Fields] OR "recurrents"[All Fields]) OR ("unresectability"[All Fields] OR "unresectable"[All Fields] OR "unresected"[All Fields])) AND 1969/01/01:2025/03/01[Date - Publication] |
| ASCO | ((((first-line) OR (previously untreated)) AND (nivolumab OR pembrolizumab OR toripalimab OR camrelizumab OR tislelizumab OR sintilimab OR serplulimab OR cemiplimab OR atezolizumab OR avelumab OR durvalumab OR (PD-1) OR (PD-L1))) AND (((gastric) OR (gastroesophageal junction)) OR (gastroesophageal junction))) AND ("1969/01/01"[Date - Publication] : "2025/03/01"[Date - Publication]) |
| ESMO | ((((first-line) OR (previously untreated)) AND (nivolumab OR pembrolizumab OR toripalimab OR camrelizumab OR tislelizumab OR sintilimab OR serplulimab OR cemiplimab OR atezolizumab OR avelumab OR durvalumab OR (PD-1) OR (PD-L1))) AND (((gastric) OR (gastroesophageal junction)) OR (gastroesophageal junction))) |
